# Mfd protects against oxidative stress in *Bacillus subtilis* independently of its canonical function in DNA repair

**DOI:** 10.1186/s12866-019-1394-x

**Published:** 2019-01-28

**Authors:** Holly Anne Martin, Katelyn E. Porter, Carmen Vallin, Tatiana Ermi, Natalie Contreras, Mario Pedraza-Reyes, Eduardo A. Robleto

**Affiliations:** 1 School of Life Sciences, University of Nevada, Las Vegas, 4505 Maryland Parkway, Las Vegas, Nevada 89154 USA; 2 Department of Biology, Division of Natural and Exact Sciences, University of Guanajuato, P.O. Box 187, Gto. 36050 Guanajuato, Mexico

**Keywords:** Mutagenesis, Oxidative damage, Mfd, MutY

## Abstract

**Background:**

Previous reports showed that mutagenesis in nutrient-limiting conditions is dependent on Mfd in *Bacillus subtilis*. Mfd initiates one type of transcription-coupled repair (TCR); this type of repair is known to target bulky lesions, like those associated with UV exposure. Interestingly, the roles of Mfd in repair of oxidative-promoted DNA damage and regulation of transcription differ. Here, we used a genetic approach to test whether Mfd protected *B. subtilis* from exposure to two different oxidants.

**Results:**

Wild-type cells survived *tert*-butyl hydroperoxide (*t-*BHP) exposure significantly better than Mfd-deficient cells. This protective effect was independent of UvrA, a component of the canonical TCR/nucleotide excision repair (NER) pathway. Further, our results suggest that Mfd and MutY, a DNA glycosylase that processes 8-oxoG DNA mismatches, work together to protect cells from lesions generated by oxidative damage. We also tested the role of Mfd in mutagenesis in starved cells exposed to *t-*BHP. In conditions of oxidative stress, Mfd and MutY may work together in the formation of mutations. Unexpectedly, Mfd increased survival when cells were exposed to the protein oxidant diamide. Under this type of oxidative stress, cells survival was not affected by MutY or UvrA.

**Conclusions:**

These results are significant because they show that Mfd mediates error-prone repair of DNA and protects cells against oxidation of proteins by affecting gene expression; Mfd deficiency resulted in increased gene expression of the OhrR repressor which controls the cellular response to organic peroxide exposure. These observations point to Mfd functioning beyond a DNA repair factor in cells experiencing oxidative stress.

**Electronic supplementary material:**

The online version of this article (10.1186/s12866-019-1394-x) contains supplementary material, which is available to authorized users.

## Background

Reactive oxygen species (ROS) are a collection of chemical species that contain one or more unpaired electrons such as superoxide, hydroxyl radical, and hydrogen peroxide [1, 2]. Oxygen radicals and non-radical oxidizing agents that can be directly modified into radicals are members of this class. Because of their reactivity, these species damage all cellular components, including nucleic acids and proteins. There are many sources of ROS in the environment [3]. However, one endogenous source is from respiration, which makes ROS inescapable sources of damage that inflict cytotoxic and genotoxic effects on cells [4, 5].

In *B. subtilis*, a bacterial model for cell growth and differentiation, the cytotoxic effects of oxidative stress are countered by regulons under the control of several transcription factors (e.g., PerR, OhrR, Spx, YodB, SigB, and MgsR); some of them are activated by sensing the redox state of the cell [6]. These regulons have been elucidated by experiments examining how cells respond to exposure to oxidants that include hydrogen peroxide, paraquat, organic peroxides, and diamide [6]. Genes that are transcriptionally active, directly or indirectly [7, 8], in these conditions code for factors that detoxify ROS (e.g., *kat* and *sod, ahpC, ahpF*), confer resistance to heavy metals (e.g., *arsB*), prevent protein misfolding (e.g., *groES*, *dnaK*), and maintain thiol-disulfide homeostasis in the cell (*trx*, *cys, bsh*) [9, 10].

On the other hand, the prevention of oxidative damage to DNA is mediated by MutT, MutM, and MutY proteins, altogether called the guanine oxidized (GO) system [11–14]. Guanine residues have the lowest reduction potential, which makes them a preferred substrate of ROS. Oxidative damage of guanines results in the formation of an 8-oxoG (OG) lesion [15]. MutT, a nucleotide sanitizer, prevents ROS damage to DNA by avoiding the incorporation of oxidized dGTPs and GTPs into nucleic acid molecules [11, 16]. MutM and MutY, DNA glycosylases, function within the pathway known as the Base Excision Repair (BER) system [17]. The MutM enzyme works as a DNA glycosylase that removes 8-oxoG from the backbone of the DNA, which is subsequently replaced with an undamaged guanine by DNA PolI [14]. If left unrepaired, 8-oxoG mispairs with adenine residues leading to transversion mutations. The MutY glycosylase preferentially targets the 8-oxoG:A pair. MutY cleaves the adenine from the sugar-phosphate backbone and produces an abasic site. Both MutM and MutY create an abasic (AP) site. AP sites are the substrate for AP endonucleases which nick the DNA generating a 3’-OH group upstream of the AP site. This reaction primes replication by a DNA polymerase which ultimately replaces the mispaired residue. Finally, the repair reaction is sealed by ligase [3, 17, 18].

In conditions in which cells are non-dividing, ROS are proposed to be intermediates in the formation of mutations that confer fitness [19]. This phenomenon is known as stationary-phase mutagenesis (SPM) [20]. One factor required for the generation of these adaptive mutations in *B. subtilis* is the protein Mfd [21]. Mfd was first identified as the Transcription Repair Coupling Factor (TRCF) which pairs the recognition of DNA damage by the RNAP to repair by the NER pathway through the UvrABCD pathway [22]. Transcription-coupled repair has been well-characterized in the context of high-fidelity DNA repair caused by UV damage. Recent findings, however, showed that Mfd promotes SPM through the interplay with multiple DNA repair pathways including the NER and BER systems [23]. This observation prompted us to determine whether Mfd is involved in repair of DNA lesions caused by exogenous ROS. Our research indicates that Mfd promotes repair of oxidative damage in *B. subtilis* and suggests that oxidative damage is a precursor to stationary-phase mutagenesis. Interestingly, we found that Mfd is involved in protecting cells against diamide, an oxidant that targets proteins. This protection was independent of its interactions with the NER and BER pathways. Altogether, these findings suggest that Mfd affects how the cell balances the potential evolutionary benefits, such as mutagenesis, and cellular detriments presented by oxidative stress.

## Results

### Mfd protects *B. subtilis* cell viability after exposure to oxidative damage

**Fig. 1:**
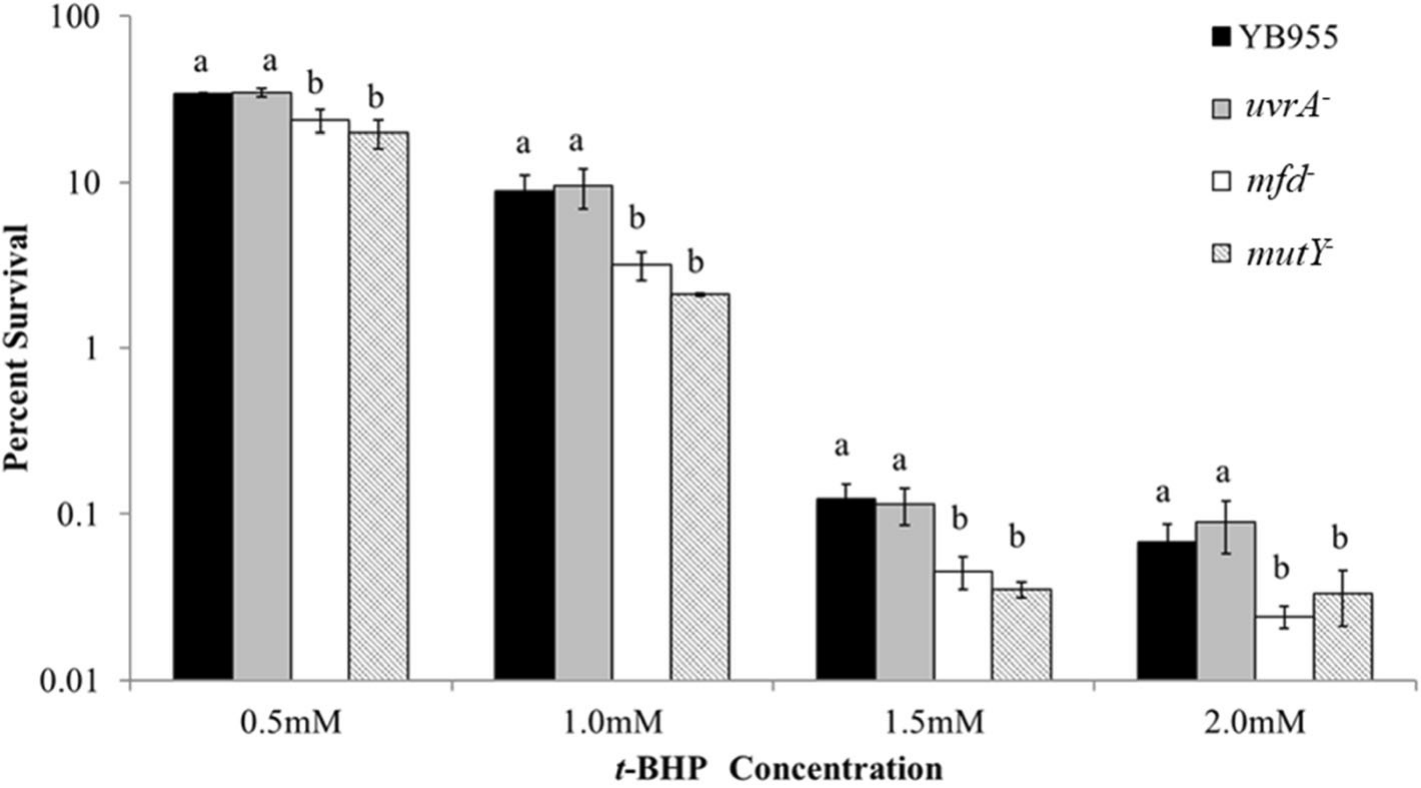
Percent cell survival, displayed on a log scale, in the parental strain (YB955) and cells deficient in the UvrA (YB9900), Mfd (YB9801), or MutY (PERM1029) respectively, following exposure to ROS via the oxidizing agent tert-*butyl* hydroperoxide (*t-*BHP). Percent survival of each strain was determined by dividing the number of colonies from each of the test concentrations by the number of colonies observed at 0 mM *t-*BHP. Means are shown for each strain. The error bars represent standard error. Means were compared using the SPSS software package and one-way ANOVA. To establish whether two means were significantly different, we used the least significant differnce (LSD) test (*P* < 0.05) between SPSS package. Lower case letters were used to denote significant differences between means. “a”, “b”, and “c” are significantly different mean groups. ANOVA and and LSD tests were conducted within each of the *t-*BHP concentrations. These experiments were replicated four times, and each replicate experiment comprise of three repetitions. The total number of observations is 12

To determine if Mfd protects against oxidative damage in stationary-phase *B. subtilis* cells, we subjected YB955 (Mfd^+^) and YB9801 (Mfd^−^) to varying levels of oxidative stress by exposing cells to the oxidant *t-*BHP. *t*-BHP is an organic peroxide that is metabolized by cells to form multiple radicals which damage several cellular components, such as lipids and DNA [6, 24]. We found that the Mfd-deficient strain had decreased survival following exposure to this oxidant agent (Fig. 1).

Because Mfd is a component of the transcription-coupled NER (UvrABCD) pathway, we tested if deficiencies in an Uvr protein also affected the ability of the *B. subtilis* cells to withstand ROS stress. The survival of all tested strains was increasingly affected by the oxidant concentration. However, only the deficiency in Mfd led to significantly lower percent survival than those observed in strains YB955 or YB9900 (UvrA^−^) (Fig. 1) and was recovered in a complemented strain (Additional file 1: Figure S1). We also tested the effects of hydrogen peroxide on cell survival as affected by Mfd and UvrA. The parental, Mfd^−^, and UvrA^−^ strains were exposed to 60 mM hydrogen peroxide for two hours during stationary phase, washed and plated to determine colony forming units. The percent survivals for YB955 and UvrA^−^ strains were similar and significantly different from the survival shown by the Mfd^−^ strain. Therefore, during the stationary phase, the absence of Mfd greatly impacted the ability of *B. subtilis* tolerate this hydrogen peroxide treatment (Additional file 2: Table S1). As a control, we tested *B. subtilis* cells for their ability to withstand UV (50 J/m^2^ UV-C); the UvrA^−^ (YB9900) strain was severely impaired in survival compared to the YB955 strain (Additional file 2: Table S1).

Because the oxidative damage protection was unaffected in the UvrA^−^ background compared to the parental strain, we tested the possibility that Mfd was interacting with a component of the GO pathway, specifically the DNA glycosylase MutY. We examined the ability of strains deficient for Mfd or MutY to the noxious effects of *t*-BHP (Fig. 1). Deficiencies in Mfd (YB9801) or MutY (PERM1029) led to significantly lower percent survival than those observed in YB955 or YB9900 (UvrA^−^). Interestingly, the survival values displayed by YB9801 (Mfd^−^) and PERM1029 (MutY^−^) were similar, which suggests that these two factors combine to work as part of a pathway to prevent oxidative damage.

**Fig. 2:**
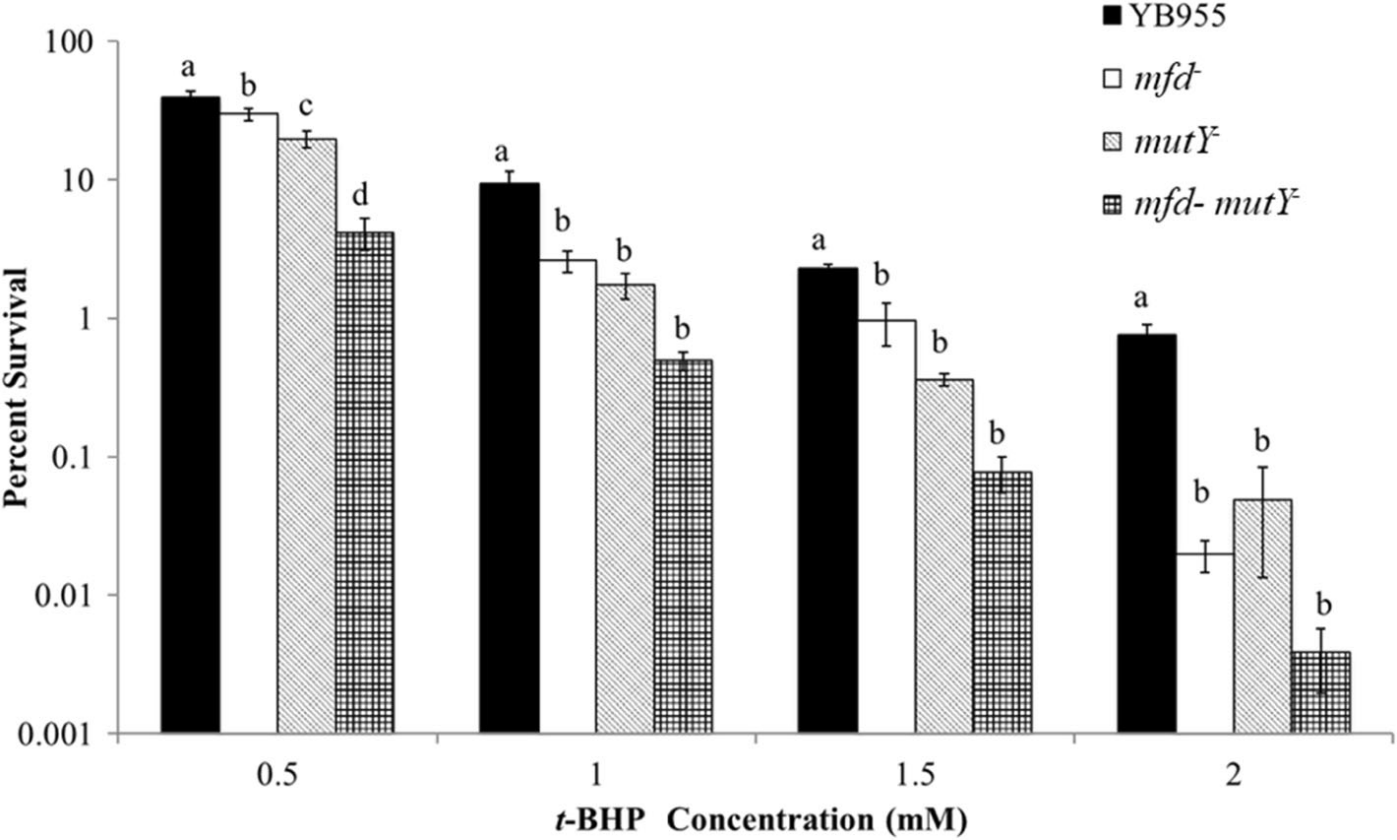
Percent cell survival, displayed in log scale, in the parental strain (YB955) and cells containing single and double defects in Mfd and MutY, (YB9801, PERM1029, and PERM818, respectively), following exposure to *tert*-butyl hydroperoxide (*t-*BHP). Percent survival of each strain was determined by dividing the number of colonies from each of the test concentrations by the number of colonies observed in the no treatment. Means are shown for each strain. The error bars represent standard error. Means were compared using the SPSS software package and one-way ANOVA. To establish whether two means were significantly different, we used the least significant differnce (LSD) test (*P* < 0.05) between SPSS package. Means with the same letter are not significnatly different. ANOVA and and LSD tests were conducted within each of the *t-*BHP concentrations. These experiments were replicated four times, and each replicate experiment comprise of three repetitions. The total number of observations is 12

We further tested the possibility that the combination of MutY and Mfd protected cells during exposure to ROS. The *t-*BHP treatment showed that single inactivation of *mfd* or *mutY* led to similar values in cell survival. Deficiencies in Mfd or MutY resulted in decreased survival compared to the parental strain (Fig. 2). Of note, the double mutant *mfd mutY* showed lower levels of survival than the strains carrying single mutations. This observation suggested that both factors protected cells against oxidative stress additively; perhaps Mfd exerted an added protection by a pathway independent of DNA repair.

### Oxidative damage leads to stationary-phase mutagenesis in a Mfd- and MutY-dependent manner

The results from the cell survival experiments suggested that Mfd and MutY act additively in preventing cytotoxicity. This observation prompted us to investigate whether Mfd and MutY were important in genotoxicity in conditions of oxidative stress. We examined how the absence of both proteins impacted mutagenesis under conditions of nutritional stress. MutY, which targets OG:A and other A mismatches, was implicated in the formation of mutations in stationary-phase cells of *B. subtilis*, particularly in the absence of the mismatch repair system [18]. Thus, we examined the possibility that oxidative stress was activating a Mfd-MutY protective system against DNA lesions that produce mutations. To measure mutagenesis, we used strains that contained an isopropyl β-D-1 thiogalactopyranoside (IPTG)-inducible, point-mutated *argF* gene in genetic backgrounds with varying proficiencies of Mfd and MutY in our stationary-phase mutagenesis assay [20]. After the cells had reached T_90_ (namely, 90 min after the cessation of growth), cultures carrying the *argF* gene construct with defects in Mfd, MutY, or both were split and placed in conditions that combine the presence and absence of IPTG (0.1 mM), and the presence and absence of *t-BHP* (1 mM) for two hours. After treatment, cells were washed twice, plated on two media: non-selective (to measure total CFU) and selective media containing IPTG and no arginine (selective for Arg^+^), and counted for CFUs at daily intervals for nine days of incubation.

**Fig. 3:**
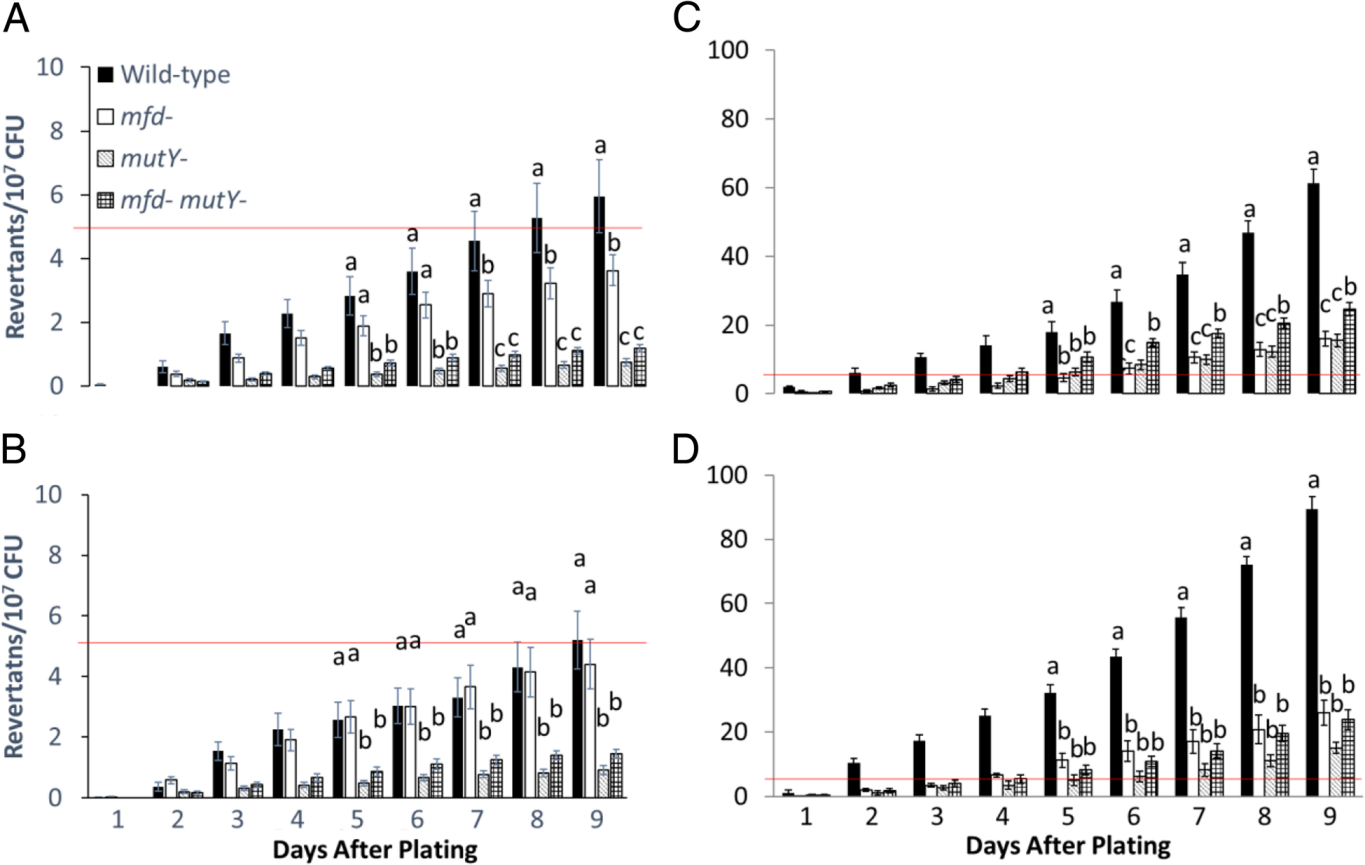
Accumulation of Arg^+^ mutations over nine days in the wild-type CV1000 strain and strains deficient in Mfd (CV1001), MutY (CV1002), or both (CV1003) when (**a**) no treatment, (**b**) induction with IPTG for two hours, (**c**) treatment with 1 mM *t*-BHP for two hours, or (**d**) induction with IPTG and treatment with 1 mM *t*-BHP for two hours. The line on each section is at the 5 revertants per 10^7^ CFU for easier comparison. Means are shown for each strain. The error bars represent standard error. Means were compared using the SPSS software package and one-way ANOVA. To establish whether two means were significantly different, we used the least significant differnce (LSD) test (P < 0.05) between SPSS package. Lower case letters were used to denote significant differences between means. “a”, “b”, and “c” are significantly different mean groups. ANOVA and and LSD tests were conducted separately for each day after plating. These experiments were replicated three times, and each replicate experiment comprise of five repetitions. The total number of observations is 15

Arg^+^ mutagenesis differed amongst the genetic backgrounds studied and ranged from 1 to 5 revertants per 10^7^ cells independently of transcriptional induction (Fig. 3; A and B, day 9). The wild-type strain and *mfd* mutant showed similar mutagenesis accumulation, but the *mutY* and *mutY mfd* mutants were notably decreased in the accumulation of Arg^+^ revertants. In cells not treated with *t*-BHP, *mutY* had greater influence than *mfd* on SPM, regardless of IPTG induction of the *argF* mutant allele.

**Fig. 4:**
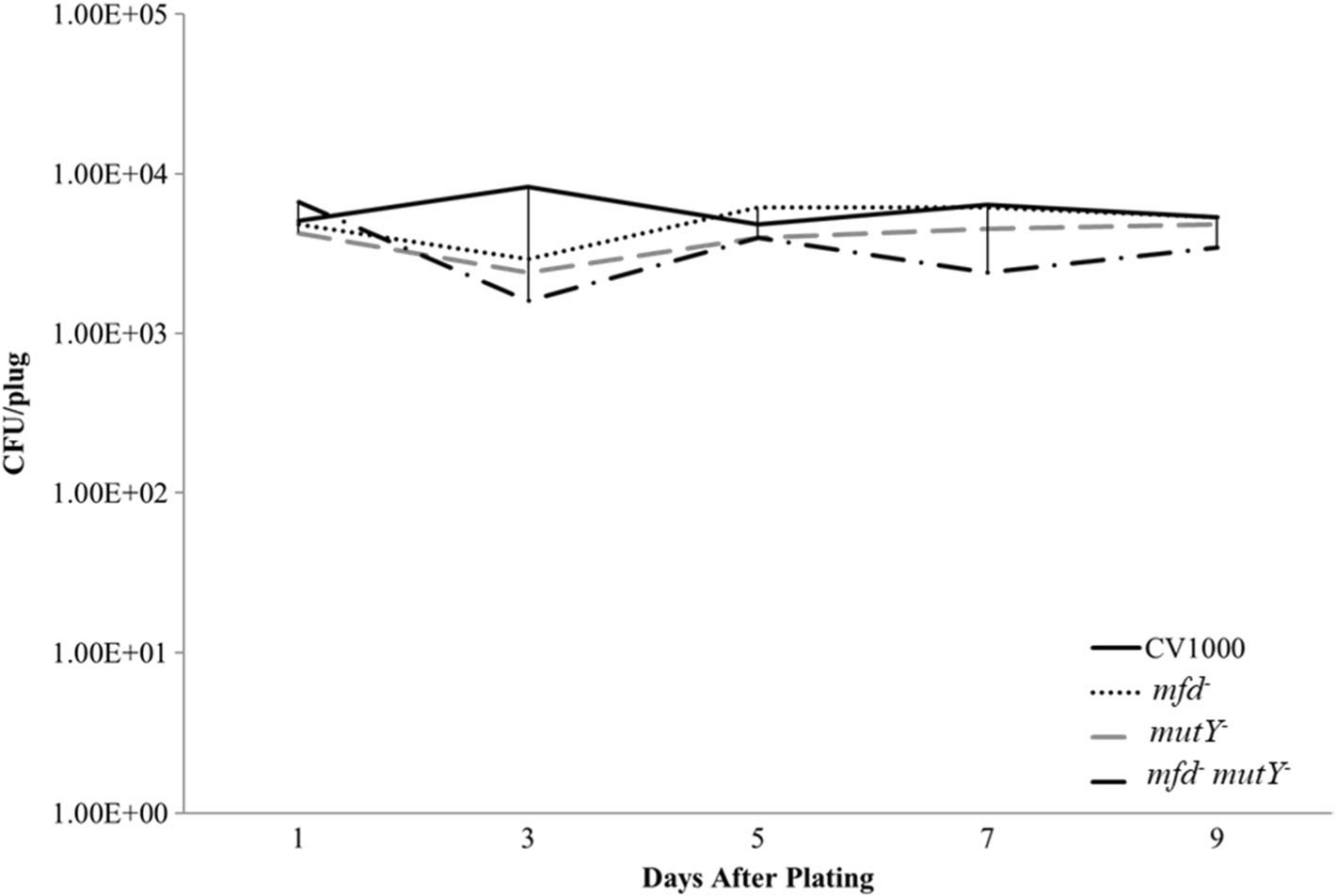
Viability of the wild-type (CV1000), Mfd-deficient (CV1001), MutY-deficient (CV1002), and double mutant (CV1003) cells over the nine-day SPM presented in Fig. 3. Procedure of how viability was measured is described in materials and methods. Each point represents an average of five samples. This is a representative figure for all four conditions (induced, uninduced, treated with *t*-BHP, and untreated with *t*-BHP)

In conditions in which cells were previously exposed to *t*-BHP, mutagenesis was strikingly increased as it ranged from 20 to 100 revertants per 10^7^ cells at day 9 (Fig. 3c). In these conditions, transcriptional induction (+IPTG) increased the number of Arg^+^ revertants produced by the wild-type strain (~ 60 to ~ 100 per 10^7^ cells at day 9) (Fig. 3d). The mutagenesis values for the single and double *mfd mutY* mutants observed in conditions of oxidative damage in the presence or absence of IPTG were similar. As controls, we conducted fluctuation assays to estimate the mutation rate of Arg^+^ prototrophy (exponentially-growing cells) as affected by oxidative stress. We found no differences (in the range of 10^− 9^ mutation per cell generated) between the treated and untreated cells of the wild-type and mutants defective in Mfd, MutY, or both (Additional file 3: Table S2). Also, a control strain carrying the inducible promoter but no *argF,* yielded no mutants (data not shown), and the viability of the cells was not affected in the different genetic backgrounds (Fig. 4). These results support the concept that Mfd and MutY participate in a mutagenic pathway that takes place in growth-limited cells experiencing oxidative stress, and that such pathway is promoted by transcriptional induction.

### Mfd, but not MutY, protects against disulfide stress

**Fig. 5:**
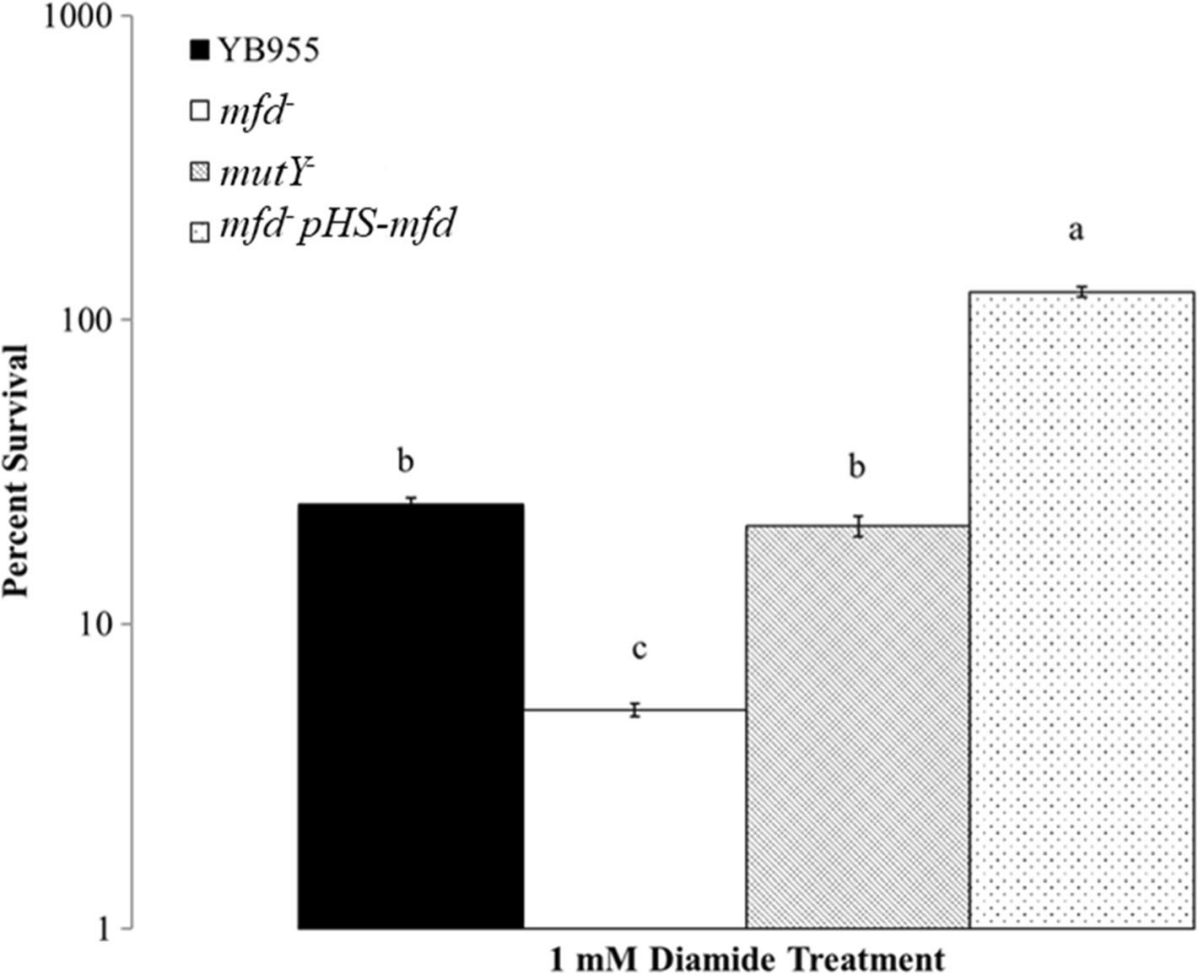
Percent cell survival, displayed in log scale, of the wild-type (YB955), Mfd-deficient (YB9801), MutY-deficient (PERM1029), and the Mfd complemented (PERM1134) strains following exposure to the oxidizing agent diamide. Percent survival for each strain was determined by dividing the number of colonies from of the test concentration by the number of colonies observed at the no treatment control. Means are shown for each strain. The error bars represent standard error. Means were compared using the SPSS software package and one-way ANOVA. To establish whether two means were significantly different, we used the least significant differnce (LSD) test (P < 0.05) between SPSS package. Lower case letters were used to denote significant differences between means. “a”, “b”, and “c” are significantly different mean groups. These experiments were replicated four times, and each replicate experiment comprise of three repetitions. The total number of observations is 12

The experiments measuring cell survival after exposure to *t*-BHP in the double knock-out (Fig. 2; Mfd^−^ and MutY^−^) suggested the possibility that Mfd may have an added effect on cell cytotoxicity in conditions of oxidative stress. We then examine whether Mfd prevented cytotoxicity against oxidative damage that targets proteins and conducted experiments that exposed cells to diamide, an oxidant that targets thiol groups and thus limits oxidative damage to proteins [25, 26]. Surprisingly, cells of the strain YB9801 (Mfd^−^) were more sensitive to the harmful effects of the protein oxidant than were the cells of the parental YB955 strain. However, cells lacking MutY (PERM1029) behaved similarly to the parental strain cells and cells containing an inducible Mfd construct survived substantially better (Fig. 5 and Additional file 4: Figure S2). Therefore, we concluded that in addition to participating in TC-NER and now documented role in oxidative damage repair, Mfd is involved in protecting cells under stress conditions that induce protein damage.

### Mutagenesis in growing cells is unaffected by Mfd

We also tested the effects of these oxidants on mutagenesis in growing *B. subtilis* cells. To this end, we determined the frequency of rifampin-resistant mutants in exponentially growing cultures exposed to no exogenous oxidant, *t*-BHP, and diamide. The mutation frequency values were statistically similar in the parental, Mfd^−^, UvrA^−^, and Mfd-restored strains (Additional file 5: Table S3). The mutation rate values for those strains in the presence and absence of oxidants ranged from a two-fold increase to a three-fold decrease compared to the untreated strain YB955. The MutY-deficient strain showed a significant increase in the mutation frequency to rifampicin resistance compared to the parent strain, even in the absence of exogenous oxidant. Of note, in the presence of either oxidant, the MutY^*−*^ strain showed further increases in the Rif^R^ mutation frequency; however, those increases were not significantly different from the untreated MutY^−^ condition. These results suggest that MutY prevents DNA lesions in growing cells. Such lesions may be produced during respiration or when cells experience oxidative stress. These results are in stark contrast to those observed in ArgF^+^ mutagenesis in stationary-phase cells. MutY was pro-mutagenic and worked in combination with Mfd, which did not affect mutation frequency in growing cells.

### Mfd affects gene expression after exposure to oxidative stress in *B. subtilis*

To better understand the role of Mfd in the response to oxidative stress, we conducted reverse transcription quantitative PCR (RT-qPCR) in cells differing in Mfd proficiency in conditions of oxidative stress. We measured gene expression of *ohrR* and *yodB*, two genes that encode for transcriptional factors which sense the redox state of the cell and derepress transcription of genes that respond to *t*-BHP and diamide [27]. In conditions of oxidative stress, these two repressors are oxidized at cysteine residues which results in derepression of genes that code for detoxifying enzymes. We also measured the *veg* gene as a control to estimate fold increase in gene expression [28]. Stationary-phase cultures were split into no treament or treatment with either *t*-BHP or diamide for 2 h, subject to RNA isolation and assayed for RT-qPCR.

**Fig. 6:**
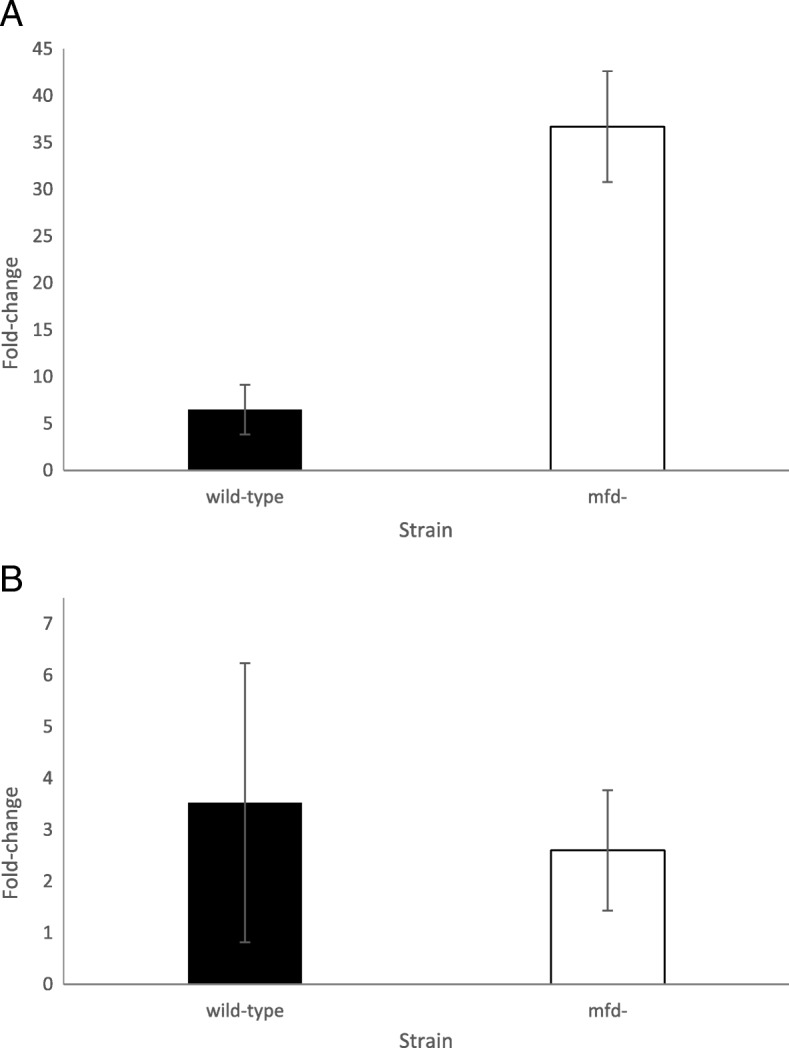
Effect of Mfd on gene expression after exposure to *t*-BHP or diamide. **a)** Fold-change in *ohrR* mRNA expression after two-hour exposure to 1 mM *t*-BHP in the parental strain (YB955) and the *mfd* mutant (YB9801). **b)** Fold-change in *yodB* mRNA expression after two-hour exposure to 1 mM diamide in the parental strain (YB955) and the *mfd* mutant (YB9801). Fold-change in expression was calculated using the 2^-ΔΔCt^ method. Means and standard error are presented. The *veg* gene was used as the control. Each condition was replicated three independent times. For **a**) and **b**), there were three technical replicates for each condition (total *n* = 9)

The wild-type and Mfd mutant cells displayed increased expression of *ohrR* in response to a two-hour exposure to *t*-BHP; however, the increase shown by the two strains was markedly different. While the wild-type cells showed a five-fold (±2.7) increase in *ohrR* expression, the Mfd-deficient cells increased *35*-fold (±5.9) the expression of this gene (Fig. 6 and Additional file 6: Table S4). The response observed in cells treated with diamide for two hours was not affected by the presence of Mfd, and both strains showed about two-and-half fold increased expression of *yodB*. In summary, the RT-qPCR results suggest that Mfd perturbs gene expression of *ohrR* in cells exposed to *t*-BHP, and that such perturbation leads to a mal-adaptation and decreased cell survival. On the other hand, the Mfd effect on cell survival after exposure to diamide did not operate through changes in expression of *yodB*, a regulator that derepresses gene expression during exposure to diamide. These results suggest that deficiencies in Mfd compromises the response to organic peroxides by increasing expression of the repressor OhrR; in contrast, Mfd may be affecting the response to disulfide stress independently of changes in expression of the YodB repressor.

## Discussion

Here, we showed that Mfd protects against *t-*BHP-generated oxidative damage in *B. subtilis*. First, the survival to *t-*BHP exposure and the Arg^+^ mutagenesis experiments revealed that Mfd and MutY cooperate to repair of *t-*BHP damage to DNA. Our data provided evidence that the role of Mfd in the processing of ROS is outside of the NER pathway. Instead, Mfd interplays with components of the BER pathway, most specifically the DNA glycosylase MutY. Further, our experiments showed that Mfd and MutY combined to form mutations, perhaps through error-prone repair [23, 29, 30]. Interestingly, *B. subtilis* cells lacking MutM were impaired in their ability to withstand treatments with hydrogen peroxide or paraquat but showed an increase in the accumulation of mutations in stationary-phase cells [14]. This response contrasts with the pro-mutagenic role of MutY observed here and elsewhere [23]. It is interesting to note that AP sites, an intermediate formed during repair of oxidative lesions, were shown to be mutagenic and cause the RNAP to stall in *E. coli* [31]. In humans, a recent report that used the SV40 immortalized cell line MRC-5 showed that oxidative damage and CS-B, a functional homolog of Mfd, recruits BER repair factors during transcription [32]. Moreover, single-stranded site gaps in Hela cells, generated during the repair of oxidative damage, blocked active RNAPII, and recruited CS-B and transcription-coupled repair [33]. In *B. subtilis,* AP sites are subject to error-prone repair that produces mutations in stationary-phase cells [29].

Because the Mfd-MutY interplay can occur in highly transcribed genes under selection and in conditions in which error-prone polymerases are expressed, one could argue that this process produces mutations with high adaptive potential. The interplay of Mfd and MutY may not require direct protein-protein interactions which are needed between Mfd and NER [23]. An indirect interaction may take place via a stalled RNAP that has encountered a MutY glycosylase processing an 8-oxo-G base pairing. In this case, a MutY occupying a lesion site represents a DNA-protein block to active RNAP elongation. Then, Mfd could dislodge the stalled RNAP thereby making the lesion available for further processing. Evidence supporting a model in which Mfd clears RNAP stalled by DNA-repressor complexes has been presented in *B. subtilis* [34–36].

One interesting aspect of the stationary-phase mutagenesis results is the prolonged effect of the transcriptional induction of the *argF* allele (two hours) and the *t*-BHP exposure (two hours) on the accumulation of mutants over 9 days. We speculate that this lasting effect on mutagenesis is the potential result of processing an overwhelming number of DNA lesions generated during the oxidant exposure. Another possibility is a slow rate of repair. Alternatively, the combination of the exposure to the oxidant and the increased transcription leads to a DNA-damage-tolerance-like cellular state. Elucidating between these possibilities ought to be the subject of future work.

The reports on the protective role of Mfd against oxidative damage are different in other experimental systems. In *E. coli* growing cells, there were no differences in survival to oxidative damage between the parental strain and Mfd mutant [37]. Contrastingly, in experiments measuring transcriptional bypass of oxidative damage in non-replicating *E. coli* cells, Mfd prevented such event [38]. In nuclear extracts from Hela cells, the CS-B factor facilitated transcriptional bypass of DNA templates containing 8-oxo-G lesions [39]. However, in vitro experiments suggest that the *E. coli* RNAP transiently pauses at such sites but does not recruit Mfd [40]. Recent biochemical evidence indicates that Mfd is important for repair of DNA lesions that are positioned remotely downstream of a paused RNAP [41]. Thus, it is possible that in stressed *B. subtilis* cells, when transcriptional profiles are very different from those observed in growing conditions and the DNA replication machinery is less active [42–45], Mfd acts as a factor that senses a wide variety of lesions. This would include those lesions that are not recognized by the NER system or not known to block the RNAP. Previous reports support the concept that Mfd is a factor that maintains genome integrity in growing cells [46, 47]. However, our experiments indicate that this factor also licenses the production of genetic diversity in times of stress, particularly when ROS and organic peroxides are increased.

The experiments using different type of oxidants lend support to the concept that Mfd protects the cell from organic peroxides by facilitating gene expression of factors that deactivate such compounds; this is unprecedented. *B. subtilis* activates different regulons to counter the effects of ROS [6]. However, our experiments focused on exposure of cells to an organic peroxide and a protein crosslinker. Exposure to diamide results in modification of the cellular proteome [48]; proteins with cysteine residues are sensitive to disulfide bond formation [49].

The results observed after treatment with diamide prompted us to examine the effect of Mfd on gene expression. Treatment with *t*-BHP results in gene derepression of *ohrA*, which codes for an organic hydroperoxide detoxifier. Transcriptional activation of *ohrA* is controlled by the repressor OhrR [10, 24, 50]. This repressor in its reduced state occludes the promoter region of *ohrA* and prevents its transcription. Oxidation of OhrR at the thiol group in residue C15 proceeds through formation of a sulfenic acid intermediate that retains DNA binding activity. However, further S-thiolation or formation of sulfonamide, sulfinic or sulfonic acid renders OhrR inactive and derepression ensues [51]. Defects in Mfd resulted in a 35-fold increase in expression of the *ohrR*. Based on these results, it is tempting to speculate that the associated increased in gene expression, observed in the absence of Mfd, precipitates an increased concentration of OhrR and a subsequent increase of the intermediate that retains repressor activity. Thus, in the presence an increased amount of active repressor, expression of detoxifying factors would be limited and lead to increased sensitivity to *t*-BHP in Mfd^−^ cells. This pattern was not observed in the experiments that exposed cells to diamide, which suggest that Mfd is affecting gene expression of factors that control thiol homeostasis independently of a transcriptional effect on YodB, a repressor that controls the response to diamide.

Our understanding of how Mfd functions has focused on DNA repair; however, there is mounting evidence indicating that its functions go beyond DNA repair. In *B. subtilis*, Mfd-deficient cells are less efficient in endospore formation [52], are affected in repression of genes that are under the controlled of catabolite repression [34, 35] or amino acid starvation [53]. Recently, single-molecule resolution experiments demonstrated that Mfd can interact with RNAP and translocate along DNA independently of its interactions with other repair proteins. Those results led the authors to postulate that Mfd modulates transcription via a catch-and-release mechanism [54]. This concept is congruent with what we observed in the experiments that exposed cells to diamide. Those experiments showed an Mfd effect that was independent of UvrA and MutY. We propose that future work use an unbiased transcriptomics approach, such as RNA-Seq, to investigate the effect of Mfd on transcription genome wide.

Furthermore, recent work has shown the importance of Mfd in the biology of multiple pathogens. Willing et al. showed that a *Clostridium difficle mfd* mutant had increased toxin production [55]. In *Staphylococcus aureus,* inactivation of Mfd was associated with decreased biofilm formation [56]. In *Helicobacter pylori*, Mfd is required for decreased sensitivity to antibiotics. Based on those observations and the results presented here, we postulate that Mfd functions as factor that protects against DNA lesions caused by oxidative damage and as a factor that modulates transcription in stressed cells. Thus, one significant implication of this study is that it proposes Mfd as a novel and attractive target to inhibit evolution of antibiotic resistance and to mitigate gene expression of bacterial virulence factors.

## Conclusions

This work indicates a role for Mfd in stressed *B. subtilis* cells tolerance to oxidative damage. First, it was demonstrated that Mfd protects against oxidative damage to DNA. Secondly, it was determined that this protection is through its interactions with MutY. Further, Mfd and MutY work cooperatively to generate mutations in stressed *B. subtilis* cells. Finally, Mfd protects against oxidative damage to proteins. The protection from the protein oxidant diamide was independent of DNA repair pathways associated with Mfd’s known functions. Thus, Mfd functions beyond transcription-coupled repair.

## Methods

### Strains

YB955 is a *B. subtilis* 168 strain containing point mutations in genes for amino acid biosynthesis of histidine (*hisC952)*, methionine (*metB5),* and leucine *(leuC427)* [20]. Genetic transformation of YB955 with antibiotic resistance gene cassettes created strains with deficiencies in Mfd, MutY, UvrA, and the double knockout Mfd/MutY. Transformations were carried out as described previously [57].

To measure mutagenesis, we used a background deficient in *argF*. A deletion of *argF* was constructed which contained a neomycin cassette. This background also included placing a point-mutated *argF* downstream of the P_hs_ promoter (IPTG-inducible) which was recombined into the *amyE* chromosomal locus. The CV1000 strain was constructed by transforming a P_hyperspank_ (pDR111) plasmid carrying the *argF* gene with a stop codon into *Bacillus subtilis.* Transformants were selected on Tryptose Blood Agar Base (TBAB) (BD, Sparks, MD) plates with 100 μg/ml of spectinomycin and screened for arginine auxotrophy. The TAA stop codon replaces the CAA codon at position 37 in the ArgF protein. The TAA stop codon was engineered into the argF sequence through PCR mutagenesis.

In brief, primer CV1000–1 was designed with an amber stop codon TAA and used in combination with WT argF reverse primer CV1000–2 carrying a SphI restriction site in PCR reactions containing Vent Polymerase. This reaction product was then used as a template for a second Vent PCR reaction using forward primer CV1000–3 and the same WT argF reverse CV1000–2 primer. This PCR product was then combined with an upstream region of argF for fusion. The two PCR products were mixed at a 1:1 ratio and all PCR reagents were added to the mixture excluding primers and GoTaq polymerase. Denaturing/annealing cycles were programmed to go on for 7 cycles to allow for hybridization of the two templates, Gotaq polymerase and primers WT argF For CV1000–4 carrying a SalI restriction site and WT argF Rev. CV1000–2 were then added to the mixture and the reaction continued for 20 cycles. The PCR products were then resolved on 1% Agarose gel and fragments corresponding to 1.2 kb size were excised out of the gel and cleaned up using the Qiagen MinElute Gel Extraction Kit (Venlo, Netherlands). The cleanup product was then digested using SalI and SphI enzymes (New England Biolabs, Ipswich, MA), ligated to the P_hyperspank_ plasmid, and transformed into *B. subtilis* as described previously [57]*.* This construct integrated into the *amyE* region of the genome as confirmed by PCR.

Strains CV1001, CV1002, and CV1003 (Mfd^−^, MutY^−^,and Mfd^−^MutY^−^, respectively) are derivatives of CV1000. These strains were constructed by transforming genomic DNA from YB9801 and PERM1029. CV1004 was constructed by transforming P_hyperspank_ plasmid into the YB955 *arg::neo* background. All strains are defined in Table 1.

**Table 1:** Strains utilized in this study

Strain Name	Construction or Reference	Genotype
YB955	Sung and Yasbin, 2002 [20]	*metB5, hisC952, leuC427*
YB9801	Ross et al*,* 2006 [21]	*metB5, hisC952, leuC427, mfd::tc*
PERM1029	Gomez et al*,* 2016 [23]	*metB5, hisC952, leuC427, mutY::em*
PERM1134	Ramírez-Guadiana et al., 2013 [52]	*metB5, hisC952, leuC427, mfd::tc, amyE::*pHS- *mfd*
PERM818	Gomez et al*,* 2016 [23]	*metB5, hisC952, leuC427, mutY::em, mfd::tc*
YB9900	Gomez et al*,* 2016 [23]	*metB5, hisC952, leuC427, uvrA::sp*
CV1000	Constructed for this work	*metB5, hisC952, leuC427, argF:: neo, amyE::*pHS- *argFSP*
CV1001	Constructed for this work	*metB5, hisC952, leuC427, argF:: neo, mfd::tc, amyE::*pHS- *argFSP*
CV1002	Constructed for this work	*metB5, hisC952, leuC427, argF:: neo, mutY::erm*, *amyE::*pHS- *argFSP*
CV1003	Constructed for this work	*metB5, hisC952, leuC427, argF:: neo, mfd::tc, mutY::erm*, *amyE::*pHS- *argFSP*
CV1004	Constructed for this work	*metB5, hisC952, leuC427, argF:: neo, amyE::*pHS

### Preparation of oxidant solutions

This oxidative stress assay required the preparation of a stock solution of the oxidizing agent. It is important to note that preparation of an oxidizer stock is affected by water quality; the metal content in the water significantly reduces the strength of the oxidant. A sterile glass bottle was acid washed using 1 N hydrochloric acid, allowed to dry, and then re-autoclaved for sterilization. The bottle was filled with DI water and autoclaved again. To make a 1 M tert-*butyl* hydroperoxide (*t-*BHP) (Sigma Aldrich, St. Louis, MO) stock solution, two sterile microcentrifuge tubes were placed on ice. 871.8 μl of the previously sterilized DI water was placed into one microcentrifuge tube. In the second separate microcentrifuge tube, 200 μl of *t-*BHP were added. From the 200 μl aliquot of *t-*BHP, 128.2 μl were transferred into the 871.8 μl of DI water.

To create a 1 M diamide (Sigma Aldrich, St. Louis, MO) stock solution, one sterile microcentrifuge tube was placed in ice and filled with 1 ml of the sterile DI water. 172 mg of diamide was transferred into the microcentrifuge tube containing the DI water and vortexed until diamide fully dissolved. It is important to note that we were careful with the use of oxidant preparations as we have observed increased variation in the response in cell survival as a function of time. We used fresh preparations from stocks that were less than 3 months-old from the date of purchase. Also, our replicate trials were conducted within one week. In spite of these efforts, we did observe variations in cell survival; however, trends in the response by a specific strain held up across experiments.

### Cell treatment with oxidants

Cells were grown in two ml of Penassay Broth (PAB) (BD, Sparks, MD) overnight in a shaking incubator at 37 °C and 250 rpm. One ml of an overnight culture was transferred into 20 ml of fresh PAB containing 20 μl of 1X Ho-Le trace elements [58] into 125 ml flasks. Growth was tracked using a spectrophotometer measuring optical density (OD_600_) (ThermoFisher Scientific, Waltham, MA). When cells reach 90 min past the onset of stationary phase (T90), two ml of culture was transferred into five, 13 mm test tube. *t-*BHP or diamide was then dispensed at different concentrations (0 mM, 0.5 mM, 1.0 mM, 1.5 mM, 2.0 mM). While being exposed to oxidants, the cells were incubating with aeration at 37 °C and 250 rpm for two hours. Following incubation, one ml was removed from each of the 13 mm test tubes, centrifuged at 13,000 rpm for two minutes, and resuspended in 1X Spizizen Minimal Salts (SMS) [59]. Cells were washed again and resuspended in 1X SMS to remove residual oxidant. The resuspended culture was serially diluted in 10-fold and 0.1 ml of the final dilution was plated onto TBAB. Plates were incubated for 24 h at 37 °C and scored for colonies to determine survival.

### Stationary phase mutagenesis assay

Cells for this assay were prepared exactly as those subjected to the survival assays. One ml of an overnight culture was inoculated into a culture flask with 20 ml of PAB supplemented with 20 μl trace elements. Cultures were grown with aeration in an incubator at 37 °C and 250 rpm to T90. Growth tracked with a spectrophotometer measuring optical density (OD_600_). 2 ml of the culture were transferred into four, 13 mm test tube. Each test tube was subjected to a specific condition of oxidative damage and transcriptional induction for two hours. Cells were then washed twice with 1X SMS.

Aliquots of 0.1 ml were spread plated in quintuplicate on Spizizen minimal medium (SMM) containing 1× SMS, 0.5% dextrose, 50 μg/ml isoleucine, 50 μg/ml glutamate, 1.5% agar (ThermoFisher Scientific, Waltham, MA), 50 μg/ml of histidine and methionine, and a 50 μg/ml leucine (Sigma-Aldrich, St. Louis, MO), with 1 mM IPTG. The plates were incubated for nine days at 37 °C. Every 24 h, were observed for the appearance of Arg^+^ colonies. To determine the number of cells plates, titers of the *B. subtilis* cultures were measured by serially diluting the resuspended culture and plating on TBAB. Titer colonies were counted after 24 h of incubation. These experiments were repeated five times and replicated three times.

To assay the non-revertant background cells, the viability of these cells was tracked over the nine-day period. Every odd day, a plug of agar was removed from a colony-free area of each of the five plates. This was done for each condition previously mentioned. The plugs were placed in 500 μl of 1X SMS, serially diluted, and plated in triplicate on SMM containing 50 μg/ml arginine, leucine, methionine, and histidine. Colonies were observed after 48 h of incubation. This stationary phase mutagenesis assay was originally described by Sung and Yasbin [20].

### Fluctuation tests

The growth-dependent mutation rate for arginine prototrophy was measured by fluctuation tests with the Lea-Coulson formula, r/m-ln(m) = 1.24 [60]. During the second overnight growth in PAB, cultures were grown in the presence or absence of 0.5 mM diamide, 0.5 mM *t*-BHP, and 1 mM IPTG. Three parallel cultures were used to determine the total number of CFU plated on each plate by titration. The mutation rates were calculated as previously described with the formula m/2Nt [20, 60].

### Quantitative real-time PCR

Cells for this assay were grown as described above. Two ml of an overnight culture was inoculated into a culture flask with 50 ml of PAB supplemented with 50 μl trace elements. Cultures were grown with aeration in an incubator at 37 °C and 250 rpm to T90. Growth tracked with a spectrophotometer measuring optical density (OD_600_). Then, the cultures were divided in half and grown for an additional two hours. One half of the culture was exposed to 1 mM *t*-BHP or diamide. Cells were then pelleted, and RNA was extracted using the RiboPure Bacteria Kit (Ambion, Carlsbad, CA). Using the One-Step SYBR GREEN RT-qPCR kit (Quanta, Biosciences, Beverly, MA), the isolated mRNA was reverse transcribed and amplified for real-time PCR. The 25 μL reactions contained the master mixes of One-Step SYBR Green RT-qPCR containing 50 ng of RNA and 300 nM final concentration of the appropriate primers (listed in Table 2). With the *veg* gene servicing as the internal control, three replicates from each culture condition were assayed and normalized [61, 62]. Reactions with no reverse transcriptase and no-template, respectively, served as controls. These reactions were run on a Bio-Rad iCycler iQ Real-Time PCR Detection System (Bio-Rad, Hercules, CA, USA), using the manufacturer’s suggested protocol and an annealing temperature of 60 °C. Results were calculated by the 2^-ΔΔCT^ (where Ct is threshold cycle) method for relative fold expression [63].

**Table 2:** Primers utilized in this study

Primer	Sequence^a^
CV1000–1	GGTGAGCTGAAA*TAA*AACAAAATTCAGCCTTAGTAT
CV1000-2	ACCAGCATGCCCTCCTTTTTGCTGTAGTAT
CV1000–3	TAAACGCCTTGCTCGCAGAAGCCGGTGAGCTGAAATAAAACAAA
CV1000–4	GATGTCGACTAAAAAGGAAGTGGCATCATGCACACAGTGACGCAA
CV1000–5	ACCAGAATGCCCTCCTTTTTGCTGTAGTATGC
F sigB	GATGAAGTCGATCGGCTCATAAG
R sigB	AACGATTTGCCGACAACAGG
F veg	GGCGAAGACGTTCGATA
R veg	CAGCTCAACAGTCTCAGTCA
F mutY	AAGGGCTCGGCTATTATTCGC
R mutY	TCCTGGACATGACACGCATC
F perR	AGGAAACCGGAGTTCGCATT
R perR	CTGCTGGAAGCATCACCGTA
F ohrR	ACAAAGCAATACAAGCCGCTG
R ohrR	GGACCGCTCATCCTCTTCAG
F yodB	GGGCCGAAACGGTTTAAAGA
R yodB	AAATTGATCGGCCCATGCCT
F mfd	GAGAAGCGAGCAAGGGCTAT
R mfd	CTTAAACGCACGTATGG

^a^Underlined sequences indicate restriction sites introduced for cloning purposes

### Statistical analysis of data

Statistical analysis was conducted with the SPSS software. Data from survival experiments testing *t*-BHP and diamide were entered in a SPSS spreadsheet using two variables. Each of the strains tested was designated as a single level within the independent variable. The values for the dependent variable consisted of the survival values. Within the analyze module of SPSS, we used the compare means program to run one-way analysis of variance (ANOVA) an α significance of *P* < 0.05. We coupled the one-way ANOVA with post hoc multiple comparisons of means. To determine whether two means were significantly different, we used the least significant difference test (LSD) at P < 0.05 in SPSS. This test calculates a critical value between two means. If the difference between two means is higher than the critical value, then the tested means are significantly different. The formula to calculate the value of the LSD is: LSD_A,B_ = (t_0.05/2 DFW_) x √((MSW) x (1/n_A_ + 1/n_B_)). The formula uses the t-distribution (two-tail), the degrees of freedom for within groups in the ANOVA table (DFW), the mean square value of the within groups in the ANOVA table (MSW), and the number of observations for each of the means (n). We used a letter system to denote significant differences between means. We assigned “a” to the means that were not significantly different from the mean with the with the highest value, “b” to means that were different from the “a” group, and so on. Data from mutagenesis experiments were processed in SPSS similarly.

## Additional files

Additional file 1: **Figure S1.** Percent cell survival of the wild-type (YB955), Mfd-deficient (YB9801), and the Mfd complemented (PERM1134) strains following exposure to ROS via the oxidizing agent *tert*-butyl hydroperoxide (1 mM *t*-BHP). Percent survival for each strain was determined by dividing the number of colonies from of the test concentration by the number of colonies observed in the no treatment control. Means and standard errors are presented. The graph shows an average of three independent trials. Each independent trial included three repetitions. (DOCX 36 kb)

Additional file 2: **Table S1.** Survival to hydrogen peroxide and UV-C in *B. subtilis* strains with defects in components of the base excision repair and the nucleotide excision repair system. (DOCX 27 kb)

Additional file 3: **Table S2**. Arg^+^ reversion rates for CV1000 (wild-type), CV1001 (Mfd^−^), CV1002 (MutY^−^), and CV1003 (Mfd^−^ MutY^−^) as affected by exposure to the oxidants *tert*-butyl hydroperoxide (*t*-BHP) or diamide. Data was analyzed using one-way ANOVA at *P* < 0.01. To determine significance between rates of mutations, we compared each rate to the untreated YB955 rate using the LSD test. There were no statistically significant differences between rates. (DOCX 15 kb)

Additional file 4: **Figure S2.** **A)** Percent cell survival, displayed in log scale, in parental cells (YB955) and cells containing a defect in Mfd (YB9801) or MutY (PERM1029) following exposure to the oxidizing agent diamide. Percent survival for each strain was determined by dividing the number of colonies from each of the test concentrations by the number of colonies observed at the no treatment control. Means are shown for each strain. The error bars represent standard error. Means were compared using the SPSS software package and one-way ANOVA. To establish whether two means were significantly different, we used the least significant differnce (LSD) test (*P* < 0.05) between SPSS package. Lower case letters were used to denote significant differences between means. “a”, “b”, and “c” are significantly different mean groups. ANOVA and and LSD tests were conducted within each of the diamide concentrations. These experiments were replicated four times, and each replicate experiment comprise three repetitions. The total number of observations is 12. **B)** Percent cell survival, displayed in log scale, in parental cells (YB955) and cells containing a defect in Mfd (YB9801) or UvrA (YB9900) following exposure to 1 mM diamide. Percent survival for each strain was determined by dividing the number of colonies from each of the test concentrations by the number of colonies observed at the no treatment control. The graph shows means and standard errors for three independent trials each independent trial included three repetitions. (DOCX 82 kb)

Additional file 5: **Table S3.** Rif^R^ mutation rates for YB955 (parental), YB9801 (Mfd^−^), YB9900 (UvrA^−^), PERM1029 (MutY^−^), and PERM1134 (Mfd complemented) as affected by exposure to oxidants *tert*-butyl hydroperoxide (*t*-BHP) or diamide. Data was analyzed using ANOVA which showed significance (P < 0.01) between treatments. To determine which means were significantly different from the control, means of treatments were compared to the YB955 control mean using the LSD test. * represents statistically significant differences between means. (DOCX 13 kb)

Additional file 6: **Table S4.** **A)** Ct values from RT-qPCR assays of veg and ohrR genes from the parental strain (YB955) and the mfd mutant strain (YB9801) with and without exposure to 1 mM t-BHP for two hours. **B)** Ct values from RT-qPCR assays of veg and yodB genes from the parental strain (YB955) and the mfd mutant strain (YB9801) with and without exposure to 1 mM diamide for two hours. (DOCX 18 kb)

## Abbreviations

ANOVA: One-way analysis of variance
AP: Abasic
BER: Base excision repair
CFU: Colony-forming units
dGTP: Deoxyguanosine triphosphate
GO: Guanine oxidized
GTP: Guanosine triphosphate
IPTG: Isopropyl β-D-1 thiogalactopyranoside
NER: Nucleotide excision repair
OG: 8-oxoG lesion
PAB: penassay broth
Rif: rifampicin
RNAP: RNA polymerase
ROS: Reactive oxygen species
RT-qPCR: Reverse transcription quantitative PCR
SMM: Spizizen minimal medium
SPM: Stationary-phase mutagenesis
TBAB: Tryptose blood agar base
*t*-BHP: *tert*-butyl hydroperoxide
TCR: Transcription-coupled repair
TRCF: Transcription repair coupling factor
UV: Ultra-violet
