# Supplementary material for: Mfd protects against oxidative stress in Bacillus subtilis independently of its canonical function in DNA repair
Source: BMC Microbiol. 2019 Jan 28;19:26. doi: 10.1186/s12866-019-1394-x (PMC6350366; doi:10.1186/s12866-019-1394-x)
Supplement: Supplementary file 1 — Figure S1. Percent cell survival of the wild-type (YB955), Mfd-deficient (YB9801), and the Mfd complemented (PERM1134) strains following exposure to ROS via the oxidizing agent tert-butyl hydroperoxide (1 mM t-BHP). Percent survival for each strain was determined by dividing the number of colonies from of the test concentration by the number of colonies observed in the no treatment control. Means and standard errors are presented. The graph shows an average of three independent trials. Each independent trial included three repetitions. (DOCX 36 kb) [file 12866_2019_1394_MOESM1_ESM.docx]

Figure S1. Percent cell survival of the wild-type (YB955), Mfd-deficient (YB9801), and the Mfd complemented (PERM1134) strains following exposure to ROS via the oxidizing agent *tert*-butyl hydroperoxide (1 mM *t*-BHP). Percent survival for each strain was determined by dividing the number of colonies from of the test concentration by the number of colonies observed in the no treatment control. Means and standard errors are presented. The graph shows an average of three independent trials. Each independent trial included three repetitions.
