# Supplementary material for: Mfd protects against oxidative stress in Bacillus subtilis independently of its canonical function in DNA repair
Source: BMC Microbiol. 2019 Jan 28;19:26. doi: 10.1186/s12866-019-1394-x (PMC6350366; doi:10.1186/s12866-019-1394-x)
Supplement: Supplementary file 2 — Table S1. Survival to hydrogen peroxide and UV-C in B. subtilis strains with defects in components of the base excision repair and the nucleotide excision repair system. (DOCX 27 kb) [file 12866_2019_1394_MOESM2_ESM.docx]

| **Table S1. Survival to hydrogen peroxide and UV-C in *B. subtilis* strains with defects in components of the base excision repair and the nucleotide excision repair system.** | |
| --- | --- |
| **A** |  |
| Strain | 60mM H_2_O_2_ |
|  |  |
| YB955 (wild-type) | 30+2% |
| YB9801 (Mfd^-^) | 1.5+2% |
| YB9900 (UvrA^-^) | 36+2% |

| **B** | |
| --- | --- |
| Strain | UV-C (50 J/m2) |
| YB955 | 26.0+1.2 |
| MutY^-^ (PERM1029) | 31.0+1.7 |
| Mfd- (YB9800) | 9.5+1.0 |
| UvrA- (YB9900) | 0.1+0.01 |

**A)** Percent cell survival of the wild-type (YB955), Mfd-deficient (YB9801), MutY-deficient (PERM1029), and UvrA-deficient (YB9900) strains following exposure hydrogen peroxide. Strain cultures were grown to T_90_, split in two aliquots, exposed to hydrogen peroxide for two hours, washed twice in an equal volume of 1X SMS, serially-diluted and plated to determine CFU. Percent survival for each strain was determined by dividing the number of colonies from of the test concentration by the number of colonies observed in the no treatment control. **B)** Percent survival to UV-C exposure. Cells were grown to T_90_. Then, 20 ml of the cell suspension were harvested, washed and resuspended in an equal volume of 1X SMS and dispensed onto a petri plate. A one-ml aliquot was extracted and serially diluted and plated on TBAB to determine CFU/ml. The remaining cells were exposed to 50 J/m^2^ of UV-C. Percent survival was estimated by dividing the number of CFU after UV-C exposure by the number of unexposed CFU. The UvrA^-^ strain (YB9900) was severely impaired in survival compared to the YB955 and MutY^-^ (PERM1029) strain. The table shows the mean and standard error for each strain. The experiments were replicated three times.
