# Supplementary material for: Mfd protects against oxidative stress in Bacillus subtilis independently of its canonical function in DNA repair
Source: BMC Microbiol. 2019 Jan 28;19:26. doi: 10.1186/s12866-019-1394-x (PMC6350366; doi:10.1186/s12866-019-1394-x)
Supplement: Supplementary file 3 — Table S2. Arg+ reversion rates for CV1000 (wild-type), CV1001 (Mfd−), CV1002 (MutY−), and CV1003 (Mfd− MutY−) as affected by exposure to the oxidants tert-butyl hydroperoxide (t-BHP) or diamide. Data was analyzed using one-way ANOVA at P < 0.01. To determine significance between rates of mutations, we compared each rate to the untreated YB955 rate using the LSD test. There were no statistically significant differences between rates. (DOCX 15 kb) [file 12866_2019_1394_MOESM3_ESM.docx]

| Strain | Condition | Mutation Rate | C.L. |
| --- | --- | --- | --- |
| CV1000 | Arg- | 5.01E-09 | 1.689E-09 |
| CV1000 | Arg- + IPTG | 6.73E-09 | 1.56E-09 |
| CV1000 | Arg- + Diamide | 5.17E-09 | 1.56E-09 |
| CV1000 | Arg- + Diamide/IPTG | 1.68E-08 | 9.16437E-10 |
| CV1000 | Arg- + *t*-BHP | 8.43E-09 | 2.2E-09 |
| CV1000 | Arg- + *t*-BHP/IPTG | 2.27E-08 | 7.3E-10 |
|  |  |  |  |
| CV1001 | Arg- | 3.81E-09 | 1.23E-09 |
| CV1001 | Arg- + IPTG | 8.41E-09 | 1.23E-09 |
| CV1001 | Arg- + Diamide | 7.87E-09 | 9.8E-10 |
| CV1001 | Arg- + Diamide/IPTG | 1.38E-08 | 1.93E-08 |
| Cv1001 | Arg- + *t*-BHP | 1.54E-08 | 1.88E-08 |
| CV1001 | Arg- + *t*-BHP/IPTG | 1.89E-08 | 1.74E-08 |
|  |  |  |  |
| CV1002 | Arg- | 5.11E-09 | 2.36E-09 |
| CV1002 | Arg- + IPTG | 3.29E-09 | 1.79E-09 |
| CV1002 | Arg- + Diamide | 3.20E-09 | 1.33E-09 |
| CV1002 | Arg- + Diamide/IPTG | 1.05E-08 | 1.56E-08 |
| CV1002 | Arg- + *t*-BHP | 4.41E-09 | 1.74E-09 |
| CV1002 | Arg- + *t*-BHP/IPTG | 1.03E-08 | 2.26E-08 |
|  |  |  |  |
| CV1003 | Arg- | 3.32E-09 | 1.33E-09 |
| CV1003 | Arg- + IPTG | 3.55E-09 | 1.50E-09 |
| CV1003 | Arg- + Diamide | 5.48E-09 | 1.41E-09 |
| CV1003 | Arg- + Diamide/IPTG | 4.08E-09 | 1.74E-09 |
| CV1003 | Arg- + *t*-BHP | 8.85E-09 | 2.00E-09 |
| CV1003 | Arg- + *t*-BHP/IPTG | 1.65E-08 | 1.97E-08 |

Table S2. Arg^+^ reversion rates for CV1000 (wild-type), CV1001 (Mfd^-^), CV1002 (MutY^-^), and CV1003 (Mfd^-^ MutY^-^) as affected by exposure to the oxidants *tert*-butyl hydroperoxide (*t*-BHP) or diamide. Data was analyzed using one-way ANOVA at P< 0.01. To determine significance between rates of mutations, we compared each rate to the untreated YB955 rate using the LSD test. There were no statistically significant differences between rates.
