# Supplementary material for: Mfd protects against oxidative stress in Bacillus subtilis independently of its canonical function in DNA repair
Source: BMC Microbiol. 2019 Jan 28;19:26. doi: 10.1186/s12866-019-1394-x (PMC6350366; doi:10.1186/s12866-019-1394-x)
Supplement: Supplementary file 4 — Figure S2. A) Percent cell survival, displayed in log scale, in parental cells (YB955) and cells containing a defect in Mfd (YB9801) or MutY (PERM1029) following exposure to the oxidizing agent diamide. Percent survival for each strain was determined by dividing the number of colonies from each of the test concentrations by the number of colonies observed at the no treatment control. Means are shown for each strain. The error bars represent standard error. Means were compared using the SPSS software package and one-way ANOVA. To establish whether two means were significantly different, we used the least significant differnce (LSD) test (P < 0.05) between SPSS package. Lower case letters were used to denote significant differences between means. “a”, “b”, and “c” are significantly different mean groups. ANOVA and and LSD tests were conducted within each of the diamide concentrations. These experiments were replicated four times, and each replicate experiment comprise three repetitions. The total number of observations is 12. B) Percent cell survival, displayed in log scale, in parental cells (YB955) and cells containing a defect in Mfd (YB9801) or UvrA (YB9900) following exposure to 1 mM diamide. Percent survival for each strain was determined by dividing the number of colonies from each of the test concentrations by the number of colonies observed at the no treatment control. The graph shows means and standard errors for three independent trials each independent trial included three repetitions. (DOCX 82 kb) [file 12866_2019_1394_MOESM4_ESM.docx]

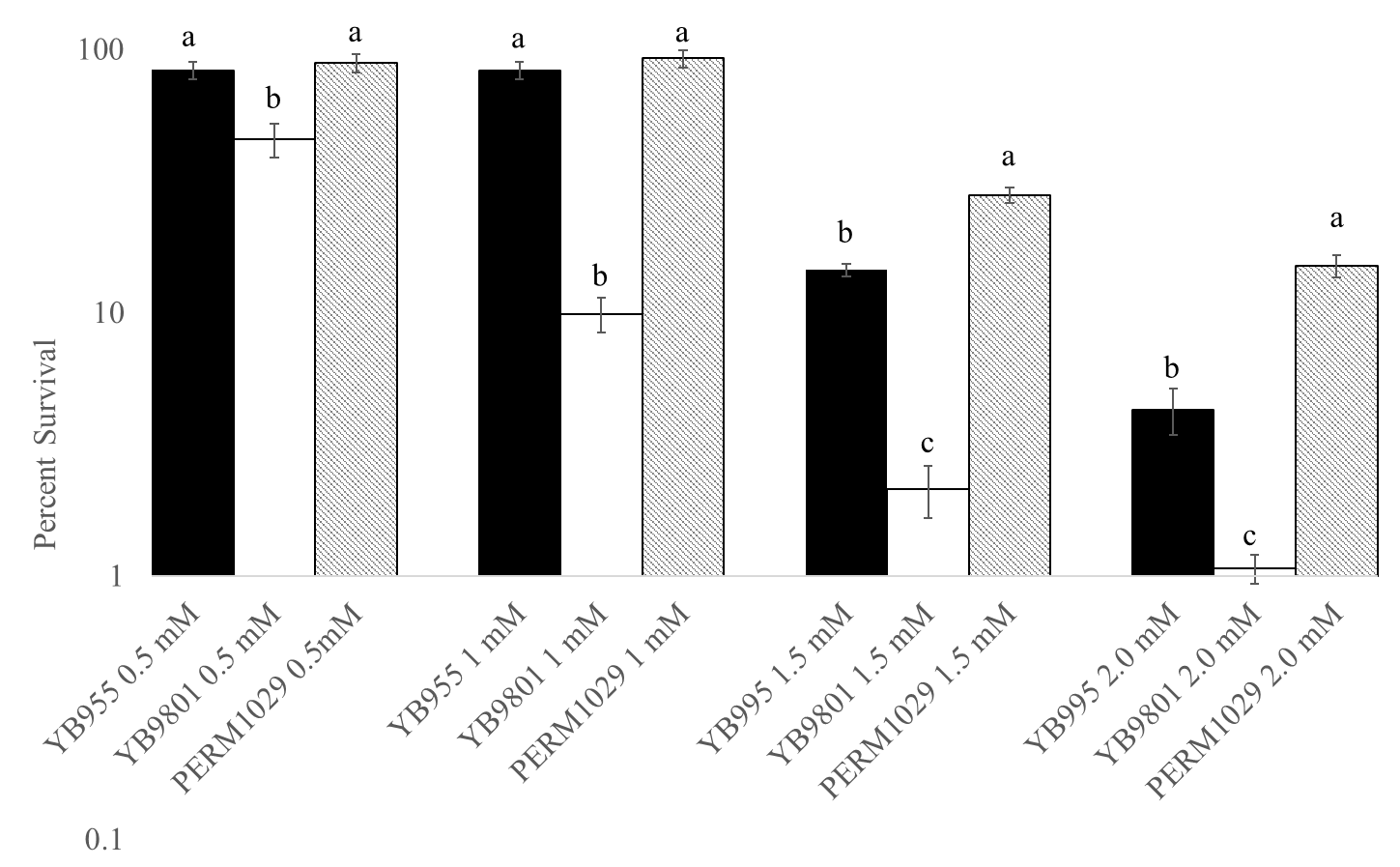


A

B

Figure S2. **A)** Percent cell survival, displayed in log scale, in parental cells (YB955) and cells containing a defect in Mfd (YB9801) or MutY (PERM1029) following exposure to the oxidizing agent diamide. Percent survival for each strain was determined by dividing the number of colonies from each of the test concentrations by the number of colonies observed at the no treatment control. Means are shown for each strain. The error bars represent standard error. Means were compared using the SPSS software package and one-way ANOVA. To establish whether two means were significantly different, we used the least significant differnce (LSD) test (P < 0.05) between SPSS package. Lower case letters were used to denote significant differences between means. “a”, “b”, and “c “are significantly different mean groups. ANOVA and and LSD tests were conducted within each of the diamide concentrations. These experiments were replicated four times, and each replicate experiment comprise three repetitions. The total number of observations is 12. **B)** Percent cell survival, displayed in log scale, in parental cells (YB955) and cells containing a defect in Mfd (YB9801) or UvrA (YB9900) following exposure to 1 mM diamide. Percent survival for each strain was determined by dividing the number of colonies from each of the test concentrations by the number of colonies observed at the no treatment control. The graph shows means and standard errors for three independent trials each independent trial included three repetitions.
