# Supplementary material for: Mfd protects against oxidative stress in Bacillus subtilis independently of its canonical function in DNA repair
Source: BMC Microbiol. 2019 Jan 28;19:26. doi: 10.1186/s12866-019-1394-x (PMC6350366; doi:10.1186/s12866-019-1394-x)
Supplement: Supplementary file 5 — Table S3. RifR mutation rates for YB955 (parental), YB9801 (Mfd−), YB9900 (UvrA−), PERM1029 (MutY−), and PERM1134 (Mfd complemented) as affected by exposure to oxidants tert-butyl hydroperoxide (t-BHP) or diamide. Data was analyzed using ANOVA which showed significance (P < 0.01) between treatments. To determine which means were significantly different from the control, means of treatments were compared to the YB955 control mean using the LSD test. * represents statistically significant differences between means. (DOCX 13 kb) [file 12866_2019_1394_MOESM5_ESM.docx]

| **Strain** | **Oxidant** | **Mutation Rate** | **Ratio to YB955 Control** |
| --- | --- | --- | --- |
| YB955 | Control | 7.05E-9±2.7 | 1.0 |
| YB955 | *t*-BHP | 4.46E-9±3.5 | 0.6 |
| YB955 | Diamide | 1.38E-8±0.4 | 2.0 |
| YB9801 | Control | 1.23E-8±0.4 | 1.7 |
| YB9801 | *t*-BHP | 5.18E-9±2.2 | 0.7 |
| YB9801 | Diamide | 9.35E-9±1.7 | 1.3 |
| YB9900 | Control | 9.84E-9±2.1 | 1.4 |
| YB9900 | *t*-BHP | 2.00E-9±0.9 | 0.3 |
| YB9900 | Diamide | 1.49E-9±0.9 | 2.1 |
| PERM1029 | Control | 1.76E-8±1.1* | 2.5 |
| PERM1029 | *t*-BHP | 3.42E-8±1.2* | 4.8 |
| PERM1029 | Diamide | 3.86E-8±1.1* | 5.5 |
| PERM1134 | Control | 1.03E-8±0.2 | 1.5 |
| PERM1134 | *t*-BHP | 5.23E-8±3.5 | 0.7 |
| PERM1134 | Diamide | 7.09E-9±2.6 | 1.0 |

Table S3. Rif^R^ mutation rates for YB955 (parental), YB9801 (Mfd^-^), YB9900 (UvrA^-^), PERM1029 (MutY^-^), and PERM1134 (Mfd complemented) as affected by exposure to oxidants *tert*-butyl hydroperoxide (*t*-BHP) or diamide. Data was analyzed using ANOVA which showed significance (P< 0.01) between treatments. To determine which means were significantly different from the control, means of treatments were compared to the YB955 control mean using the LSD test. * represents statistically significant differences between means.
