# Supplementary material for: Mfd protects against oxidative stress in Bacillus subtilis independently of its canonical function in DNA repair
Source: BMC Microbiol. 2019 Jan 28;19:26. doi: 10.1186/s12866-019-1394-x (PMC6350366; doi:10.1186/s12866-019-1394-x)
Supplement: Supplementary file 6 — Table S4. A) Ct values from RT-qPCR assays of veg and ohrR genes from the parental strain (YB955) and the mfd mutant strain (YB9801) with and without exposure to 1 mM t-BHP for two hours. B) Ct values from RT-qPCR assays of veg and yodB genes from the parental strain (YB955) and the mfd mutant strain (YB9801) with and without exposure to 1 mM diamide for two hours. (DOCX 18 kb) [file 12866_2019_1394_MOESM6_ESM.docx]

| **A** | veg |  |  |  |  |  | ohrR |  |  |  |
| --- | --- | --- | --- | --- | --- | --- | --- | --- | --- | --- |
| Sample | Ct1 | Ct2 | Ct3 | Avg. Ct |  | Sample | Ct1 | Ct2 | Ct3 | Avg. Ct |
| YB955 1 | 14.15 | 14.21 | 14.24 | 14.18 |  | YB955 1 | 22.13 | 22.49 | 22.75 | 22.13 |
| YB955 1* | 10.96 | 11.37 | 11.52 | 11.28 |  | YB955 1* | 17.84 | 18.03 | 18.42 | 18.09667 |
| YB955 2 | 13.1 | 13.25 | 12.45 | 12.93 |  | YB955 2 | 23.04 | 22.93 | 22.76 | 22.91 |
| YB955 2* | 11.68 | 11.51 | 11.9 | 11.70 |  | YB955 2* | 19.02 | 18.94 | 19.36 | 19.10667 |
| YB955 3 | 11.34 | 11.48 | 12.05 | 11.41 |  | YB955 3 | 23.17 | 23.18 | 23.12 | 23.17 |
| YB955 3* | 11.56 | 11.35 | 11.42 | 11.44 |  | YB955 3* | 19.45 | 19.89 | 19.76 | 19.7 |
| YB9801 1 | 10.8 | 10.66 | 10.75 | 10.74 |  | YB9801 1 | 22.43 | 22.43 | 22.31 | 22.39 |
| YB9801 1* | 13.25 | 13.88 | 13.35 | 13.49 |  | YB9801 1* | 20.41 | 20.09 | 19.69 | 20.06333 |
| YB9801 2 | 9.89 | 9.29 | 9.98 | 9.59 |  | YB9801 2 | 22.21 | 22.08 | 23.31 | 22.21 |
| YB9801 2* | 10.2 | 10.32 | 11.13 | 10.55 |  | YB9801 2* | 17.14 | 17.33 | 18.28 | 17.58333 |
| YB9801 3 | 11.26 | 11.73 | 11.31 | 11.43 |  | YB9801 3 | 24.29 | 24.08 | 24.26 | 24.21 |
| YB9801 3* | 14.02 | 14.34 | 13.85 | 14.07 |  | YB9801 3* | 22.65 | 21.78 | 21.67 | 22.03333 |
| *indicates exposure to *t*-BHP | | | |  |  |  |  |  |  |  |

| **B** | veg |  |  |  |  | yodB |  |  |  |
| --- | --- | --- | --- | --- | --- | --- | --- | --- | --- |
| Sample | Ct1 | Ct2 | Ct3 | Avg. Ct | Sample | Ct1 | Ct2 | Ct3 | Avg. Ct |
| YB955 1 | 20.45 | 21.23 | 19.73 | 20.47 | YB955 1 | 27.98 | 30.55 | 27.29 | 28.61 |
| YB955 1* | 19.89 | 16.54 | 17.98 | 18.14 | YB955 1* | 26.69 | 28.62 | 29.6 | 28.30 |
| YB955 2 | 20.44 | 22.95 | 25.53 | 22.97 | YB955 2 | 36.47 | 31.27 | 32.1 | 33.28 |
| YB955 2* | 21.13 | 18.33 | 18.34 | 19.27 | YB955 2* | 29.79 | 28.35 | 29.03 | 29.06 |
| YB955 3 | 22.1 | 21.05 | 20.31 | 21.15 | YB955 3 | 30.00 | 26.91 | 35.06 | 30.66 |
| YB955 3* | 17.53 | 17.57 | 19.24 | 18.11 | YB955 3* | 24.95 | 24.77 | 23.67 | 24.46 |
| YB9801 1 | 16.31 | 16.49 | 16.05 | 16.28 | YB9801 1 | 25.58 | 25.67 | 26.08 | 25.77 |
| YB9801 1* | 14.81 | 13.49 | 14.81 | 14.37 | YB9801 1* | 26.43 | 22.44 | 22.97 | 23.95 |
| YB9801 2 | 15.66 | 16.33 | 15.68 | 15.89 | YB9801 2 | 24.82 | 26.24 | 25.37 | 25.48 |
| YB9801 2* | 15.86 | 17.11 | 14.63 | 15.87 | YB9801 2* | 22.13 | 21.35 | 26.04 | 23.17 |
| YB9801 3 | 17.5 | 17.67 | 18.47 | 17.88 | YB9801 3 | 27.39 | 25.06 | 24.99 | 25.81 |
| YB9801 3* | 19.35 | 17.79 | 19.48 | 18.87 | YB9801 3* | 24.69 | 26.34 | 26.39 | 25.81 |
| *indicates exposure to diamide | | | |  |  |  |  |  |  |

Table S4. **A)** Ct values from RT-qPCR assays of *veg* and *ohrR* genes from the parental strain (YB955) and the *mfd* mutant strain (YB9801) with and without exposure to 1 mM *t*-BHP for two hours. **B)** Ct values from RT-qPCR assays of *veg* and *yodB* genes from the parental strain (YB955) and the *mfd* mutant strain (YB9801) with and without exposure to 1 mM diamide for two hours.
